# Complete Genome Sequence of Probiotic *Lactobacillus plantarum* Strain FMNP01, Isolated from Mango Fruit

**DOI:** 10.1128/genomeA.01207-14

**Published:** 2014-11-26

**Authors:** Xue-Fei Li, Xue-Yi Liao, Yong-Feng Liu, Li-Qiong Guo, Zhi-Wei Ye, Jun-Fang Lin

**Affiliations:** a Institute of Food Biotechnology, South China Agricultural University, Guangzhou, China; b Department of Bioengineering, College of Food Science, South China Agricultural University, Guangzhou, China; c BGI-Shenzhen, Shenzhen, China

## Abstract

*Lactobacillus plantarum* strain FMNP01 is a new strain with probiotic properties that was isolated from fresh mango from Guangzhou, China. Here, we report the complete genome of this organism.

## GENOME ANNOUNCEMENT

Lactic acid bacteria (LAB) have a long history of use in the food industry and usually are regarded as generally safe food starters. As one of the most impotent LAB, *Lactobacillus plantarum* is widely distributed in plant-derived foods such as fruits, pickles, wine, and bean sauces. *L. plantarum* is also considered to be one of the most important probiotics because some *L. plantarum* strains possess a variety of health benefits such as immune enhancement (1–3). We sequenced the genome of *L. plantarum* strain FMNP01, which was originally isolated from fresh mango, using shotgun strategy with Illumina HiSeq2000. Here, we report the genome sequence of *L. plantarum* strain FMNP01.

Whole-genome sequencing of *L. plantarum* strain FMNP01 was performed (BGI-Shenzhen, Shenzhen, China) with a shotgun strategy. A total of 513 Mb of next-generation Illumina paired-end 90-bp reads were generated by sequencing genome shotgun libraries of different fragment lengths (500 bp and 6 kb) with 155-fold coverage of the genome. The assembly, performed by SOAPdenovo version 1.05 (4) with optimal assembly acquired with a key parameter K value of 39, consisted of 18 contigs and 4 scaffolds.

The *L. plantarum* strain FMNP01 genome comprises a single circular chromosome (3,313,644 bp), with an overall G+C content of 44.48%. Coding sequence (CDS) prediction was carried out with Glimmer version 3.0 (5) and provided 3,147 CDSs covering 83.70% of the genome. Non-coding RNA was predicted by rRNAmmer 1.2 (6), tRNAscan-SE 1.2 (7), and Rfam 10.1 (8). There are 3,147 protein-coding genes, 16 rRNAs, 64 tRNAs, 70 minisatellite DNAs, and 3 microsatellite DNAs in the genome of *L. plantarum* strain FMNP01.

Compared with *L. plantarum* strain WCFS1 (9, 10) and *L. plantarum* strain 16 (11), *L. plantarum* FMNP01 has more than 200 unique genes. These include glycosyl transferase, acetoin utilization protein, melibiose operon regulatory protein, glycosyl hydrolase, cellulose synthase, and other proteins related to carbohydrate metabolism. Interestingly, two potassium-transporting ATPase subunit proteins, involved in inorganic ion transport and metabolism, were uniquely identified in FMNP01. Furthermore, many prophage sequences and locations of FMNP01 are novel.

In conclusion, the comparative analysis revealed that FMNP01 changed its genome to adapt the environmental niches, and the more than 200 unique genes may give it different abilities to utilize carbohydrates, transport potassium, and so on. The genome sequence gives us a basis to further elucidate the functional mechanisms of its probiotic properties.

### Nucleotide sequence accession numbers.

This whole-genome shotgun project has been deposited at GenBank under the accession number JPSU00000000. The version described in this paper is version JPSU01000000.
